# Analysis of the Impact of Public Services on Residents’ Health: A Spatial Econometric Analysis of Chinese Provinces

**DOI:** 10.3389/ijph.2023.1605938

**Published:** 2023-07-27

**Authors:** Wei Jia, Lei Liu, Zhihao Wang, Gang Peng

**Affiliations:** ^1^ School of Politics and Public Administration, Qingdao University, Qingdao, China; ^2^ School of Statistics, Southwestern University of Finance and Economics, Chengdu, China

**Keywords:** public services, residents’ public health, spatial effect, spatial Durbin model, composite indicator

## Abstract

**Objectives:** The aim of this study was to explore the mechanism between public services and residents’ health, focusing on the role of spatial geographical factors.

**Methods:** Leveraging a comprehensive panel dataset encompassing 30 mainland Chinese provinces from 2007 to 2019, this study engineered a spatial Durbin model furnished with dual fixed effects through the application of the Lagrange multiplier, Hausman, and likelihood ratio tests. The primary objective was to delve into the repercussions of varying public service levels on residents’ health outcomes.

**Results:** The empirical findings reveal a palpable spatial autocorrelation between residents’ health outcomes and the public services levels dispensed across Chinese provinces. Intriguingly, an elevation in the public service level in a given province not only ameliorates its residents’ health outcomes but also triggers a spatial spillover effect, thereby positively influencing residents’ health in neighboring provinces. The rigorous endogeneity and robustness checks affirm the reliability of the principal outcomes.

**Conclusion:** Due to the increase in social uncertainty, all regions should break free of the administrative monopoly, enhance regional integration and development, and improve residents’ health status by clustering public service supply.

## Introduction

The World Health Organization reveals that from 2000 to 2019, the average life expectancy has increased from 66.8 to 73.3 years [1]. Although the global population’s overall health status has improved, it still faces numerous risks. Social factors, such as culture [2], environment [3], and social security [4], play a significant role in population health. However, as societal uncertainties increase (such as the COVID-19 pandemic), investigating the linkage between single social factors and health may not adequately meet the growing population’s health needs; therefore, scholars have begun to focus on the impact of comprehensive public services on health [5, 6].

Public services encompass various social factors, such as education, healthcare, and infrastructure. Unraveling the intrinsic interaction mechanism between public services and residents’ health has become a focal point in health economics [7]. Public health services have been extensively explored as determinants of societal health. In China, the public health system includes health services at all levels and provides comprehensive coverage from the community, rural (grassroots health service institutions), county, city, and provincial levels. However, given the influence of regional area and differences in the allocation of central government resources, there are disparities among the different provinces. This directly results in heterogeneity in residents’ health on spatial and social dimensions [8, 9]. Further, China’s long-standing economic development-centered social governance has caused public service provision to lag behind economic growth, leading to spatial imbalances in comprehensive public service supply, not just in the realm of public health services [10].

Educators believe that unlike other social factors, education is a key factor that cannot be ignored because of its ability to provide benefits at the logical level of thinking and improve population health [11]. They posit that individuals with higher education levels will exhibit healthier lifestyle habits and gain access to more health information, thereby resulting in better health outcomes [12, 13]. Unlike the complex effects of educational services, environmental sanitation services have a more direct linear relationship with residents’ health. In the 1980s, scholars included environmental pollution as an influencing factor of population health. Although this period saw a series of debates on research models, measurement bias, and endogeneity, it remains indisputable that environmental pollution does weaken population health [14, 15]. Moreover, infrastructure and social security are also important directions for the study of public services and social health.

Collectively, the extant research has primarily investigated residents’ health from the perspective of specific public services but has rarely utilized spatial econometric methods. Given the current body of research, the assumption of spatial independence between one region and another is divergent from reality. To address these gaps, this study, based on panel data from 30 provinces in China, uses a comprehensive public service perspective to focus on the role of spatial factors in the relationship between public services and resident health, and the spatial spillover benefits of public services. The contributions of this study manifest in several ways. First, this study constructs an evaluation index system based on China’s official public service standards, thereby rendering a comprehensive index of public services closer to actual development levels. Second, by utilizing non-parametric statistics and spatial correlation tests, this study better comprehends spatial clustering aspects of resident health and public services in China. Finally, this study’s research perspective expands into regional economics and spatial statistics by employing spatial spillover econometric methods to analyze the influence relationship between public services and resident health.

## Methods

### Index Construction and Selection

#### Explained Variable: Resident Health (RH)

Public health science bifurcates human health into two principal dimensions: physiological and psychological health. Regarding physiological health, the European Community Health Monitoring Programme (HMP) has established relatively unified health measurement indicators that have been widely acknowledged by academia. Based on the HMP, Freitas et al. collected authoritative expert opinions using the Delphi selection method to further optimize the population health assessment index system [16]. During the data collection, the current study referred to the multivariate health assessment system established by Freitas et al., combined with the availability of related data, to determine six variables as indicators that reflected residents’ physiological health. When selecting the indicators for the psychological health dimension, this study, based on Yue et al. and psychological counselor work [17], chose the psychiatric consultation rate as the macro-measurement indicator for residents’ mental health level. Moreover, all resident health evaluation indicators selected in this study were “negative,” meaning that an increase in the values of each indicator corresponded to a decline in residents’ actual health level.

#### Explanatory Variable: Public Services (PS)

In 2021, the Chinese government released the “National Basic Public Service Standards (2021 version)” (hereafter Standards) to officially define the scope and development standards of basic public services [18]. This document meticulously established a development index system of basic public services in nine key areas and also defined development indices for each area. Due to the difficulty in accessing data for certain indices (such as military services), modifications were required in accordance with the standards.

This study merged the areas of early childhood, employment, older adult care, and support for the vulnerable into social security, so as to reflect the level of social welfare-type public services. The evaluation system for public housing services is complex, and current official Chinese data are unable to accurately measure its development level. Therefore, following Liu et al., this study divided public housing services into infrastructure and environmental protection [19]. Regarding military services, the current data are fragmented, lack systematic official statistics, and are thus unable to meet the public service measurement needs. Accordingly, this study did not include it in the index system. Based on the above, this study evaluated public services from six core dimensions: healthcare, social security, infrastructure, environmental protection, elementary education, and culture sports.

This study based its selection of secondary indicators on the secondary indicator system in the “Standards” and used “capacity indicators” to reflect the development level of basic public services in various fields [20]. Variables involving “*per capita*,” “per 1,000 people,” “per 10,000 people,” and “per 100,000 people” utilized the ratios of the relevant data to the population at the end of the year. To maintain data uniformity, this study calculated the population at the end of the year of each year based on the latest standards. Moreover, the public service evaluation indicators were all “positive”; that is, an increase in the value of each indicator represented a rise in the actual public service levels.

#### Control Variables

Economic level (regional *per capita* gross domestic product [GDP]): Most related studies have used regional *per capita* GDP as a proxy variable to reflect the economic development of a certain region. This study deflated the *per capita* GDP with the base year of 2000.

Urbanization level (urban population ratio): This study used the proportion of the urban population within each province to capture the regional urbanization level [21].

Human capital (proportion of the population with higher education levels): This study defined residents with tertiary (or higher) educational attainment within each province as those with higher education levels, and used the proportion of this population as the specific proxy variable for human capital [22].

### Data Collection and Extraction

The scope of this study encompasses 30 provinces (cities, districts) in mainland China, excluding Tibet, with collected data spanning from 2007 to 2019 serving as the sample [23]. All data were sourced from the China Statistical Yearbook, China Health Statistical Yearbook, China Education Fund Statistical Yearbook, China Environmental Statistical Yearbook, and China Labor Statistical Yearbook.

There are numerous methods to comprehensively evaluate residents’ health and public service levels, the entropy method is one of the mainstream assessments in objective weight assignment comprehensive evaluation. It is based on information entropy and determines the weight of each indicator variable in an evaluation system according to the degree of variation to objectively reflect differences between indicators [24]. Further, the entropy method is not affected by subjective factors and the results are stable, consistent, and can accurately reflect residents’ health and public service levels. Based on the above analysis, after obtaining the initial data, this study used the entropy method and cumulative method to calculate the weight of each indicator. To guard against bias in the regression results due to heteroscedasticity, this study performed a logarithmic transformation on *per capita* GDP (Table 1).

**TABLE 1:** Comprehensive index system and weights of residents’ health and public services (China, 2007–2019).

	Dimensional layer	Indicator layer	Unit	Attribute	Weight
Residents’ Health	Physical Health	Perinatal mortality	%	−	0.0800
		Low-weight prevalence rate of children under 5 years	%	−	0.0697
		Maternal mortality rate (1/100,000)	%	−	0.0574
		Regional population mortality	‰	−	0.3120
		Annual hospitalization rate	%	−	0.2602
		Average annual number of medical visits		−	0.1534
	Mental Health	Psychiatric consultation rate	%	−	0.0672
Public Services	Healthcare	Number of health institutions per 10,000 people		+	0.0267
		Number of health beds per 10,000 people		+	0.0313
		Number of health technicians per 10,000 people		+	0.0263
	Social Security	Coverage rate of pension insurance for urban employees (including retirees)	%	+	0.0374
		Coverage rate of medical insurance for urban employees (including retirees)	%	+	0.0607
		Coverage rate of regional unemployment insurance	%	+	0.0833
	Infrastructure	Rough Internet penetration rate	%	+	0.0697
		Road area *per capita*	Square meters	+	0.0242
		Road mileage per 1,000 km	km	+	0.0484
		Electricity consumption *per capita*	kWh	+	0.0647
		Water consumption *per capita*	Tons	+	0.1022
	Environmental Protection	Per capita domestic waste clearance volume	Tons	+	0.0548
		Utilization rate of industrial solid waste	%	+	0.0258
		Urban sewage treatment rate	%	+	0.0096
	Elementary Education	Number of primary schools per 10,000 people		+	0.0552
		Number of junior high school per 10,000 people		+	0.0259
		Public budget expenditure per pupil	¥	+	0.0543
		Public budget expenditure per junior high school student	¥	+	0.0639
		Primary school teacher–student ratio	%	−	0.0136
		Junior high school student ratio	%	−	0.0164
	Culture Sports	Number of public libraries per 100,000 people		+	0.0614
		Number of museums per 100,000 people		+	0.0441

## Results

### Spatial Correlation Test

Before conducting research using spatial econometric models, it’s necessary to perform spatial autocorrelation tests on residents’ health and public services separately, using Moran’s Index (Moran’s I) [25].

$${Moran}^{\prime }s\;I=\frac{\sum_{i=1}^{n}\sum_{j=1}^{n}W_{ij}\left(Y_{i}-\bar{Y}\right)\left(Y_{j}-\bar{Y}\right)}{S^{2}\sum_{i=1}^{n}\sum_{j=1}^{n}W_{ij}}$$ (1)

$$I_{i}=\frac{\sum_{j\neq i}^{n}W_{ij}\left(Y_{i}-\bar{Y}\right)\left(Y_{j}-\bar{Y}\right)}{S^{2}}$$ (2)

The calculated Moran’s I value is within the interval [−1, 1], bounded by zero. The positive and negative values indicate positive spatial autocorrelation and negative spatial autocorrelation of the variables, respectively, and larger values represent stronger correlations. If the result is zero, then the variables are spatially irregular and should not be studied using spatial econometric models. Moreover, this study constructed the adjacency spatial weight matrix according to whether the provinces were adjacent. Two provinces with adjacent administrative partition lines were denoted as one, and zero if otherwise.

#### Global Spatial Autocorrelation Test

The contents of Table 2 demonstrate the results of the Global Moran’s I. The public services in each Chinese province display positive spatial correlations from 2007 to 2019 and all Z statistics are significant, indicating robust positive spatial correlation. The Moran’s I outcomes suggest that the spatial correlation of public services displays a downward trajectory in the time series, which might be associated with society’s digital transformation. The results in Table 2 reveal that residents’ health among the Chinese provinces has a strong and positive spatial correlation. Regarding the temporal variation, the Moran’s I value for residents’ health relatively oscillates and generally demonstrates a “wave-like” decline, reducing the spatial dependence. In sum, both residents’ health and public services display spatial autocorrelation, which validates the feasibility of using spatial econometric models to test the mechanism between public services and residents’ health in this study.

**TABLE 2:** Global Moran’s index of residents’ health and public services (China, 2007–2019).

Year	Moran’s index (RH)	Z (RH)	Moran’s index (PS)	Z (PS)
2007	0.3415	3.1034***	0.3615	3.2746***
2008	0.3246	2.9600***	0.3608	3.2705***
2009	0.3998	3.5572***	0.3621	3.2824***
2010	0.3123	2.8427***	0.3599	3.2720***
2011	0.2765	2.5516**	0.3596	3.2707***
2012	0.3210	2.9193***	0.3165	2.9242***
2013	0.3331	3.0068***	0.2905	2.7150***
2014	0.3462	3.1181***	0.2635	2.4887**
2015	0.3031	2.7631***	0.2290	2.1989**
2016	0.2587	2.3943**	0.2206	2.1248**
2017	0.2658	2.4573**	0.2263	2.1750**
2018	0.2478	2.3155**	0.1876	1.8636*
2019	0.2932	2.6835***	0.1866	1.8561*

Note: ***, **, and * indicate 1%, 5%, and 10% significant levels, respectively.

#### Local Spatial Autocorrelation Test

**FIGURE 1:**
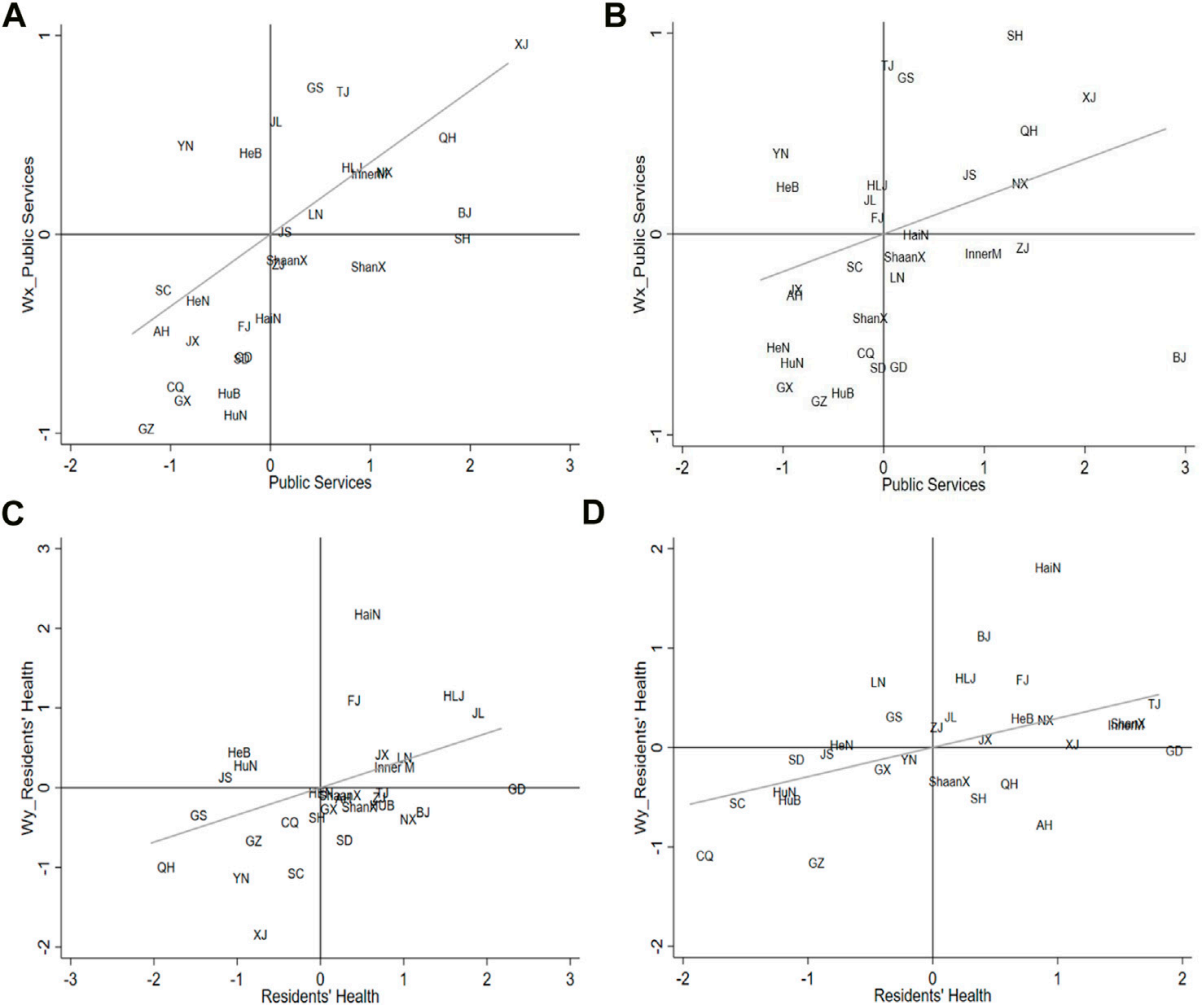
Moran’s Index scatter diagram of public services and residents’ health (China, 2007–2019): **(A)** Moran’s Index scatter plot of public services in China, 2007; **(B)** Moran’s Index scatter plot of public services in China, 2019; **(C)** Moran’s Index scatter plot of residents’ health in China, 2007; **(D)** Moran’s Index scatter plot of residents’ health in China, 2019.

This study, based on data from 2007 to 2019, conducted a local spatial autocorrelation analysis of residents’ health and public services (Figure 1), thereby examining the spatial aggregation and development changes from temporal and spatial perspectives [26].

Regarding public services, most Chinese provinces are located in the first and third quadrants; that is, “high–high” and “low–low” clustering areas, respectively. By 2019, the number of provinces in the second and fourth quadrants increases and the positive spatial correlation of public services in each province weakens. The spatial clustering status of residents’ health in each province varies greatly over the time series. In 2007, the vast majority of provinces are distributed in the first, third, and fourth quadrants, mainly forming “high–high,” “low–low,” and “high–low” clustering areas. By 2019, the number of provinces in the first and third quadrants had noticeably increased, with their proportion rising from 53.3% to 83.3%. Therefore, it can be inferred that the spatial clustering degree of residents’ health in each province in China is gradually increasing, but the differentiation degree between different clusters is also relatively obvious. In summary, both residents’ health and public services in the current provinces exhibit clustering states of “high–high” and “low–low.”

**FIGURE 2:**
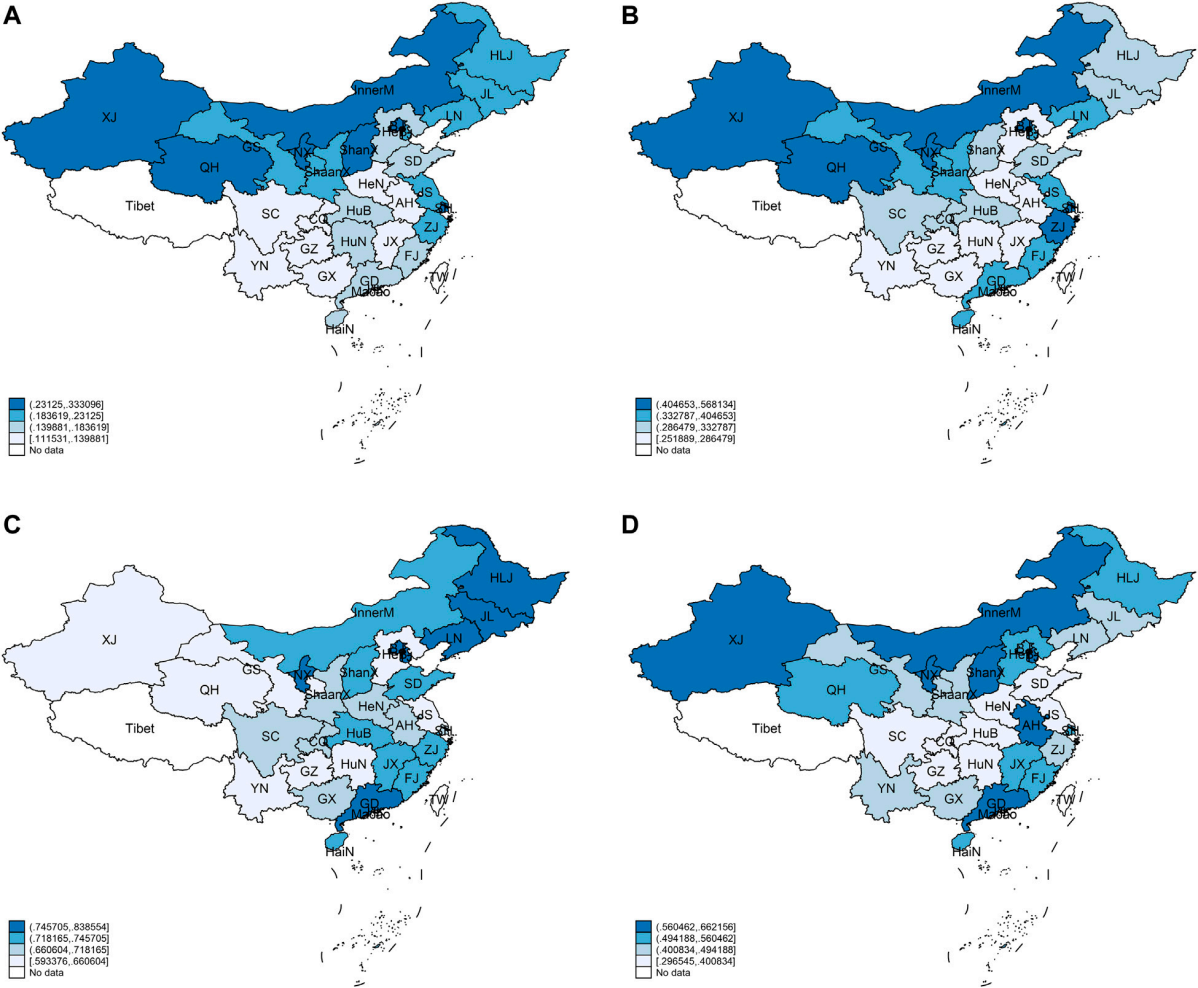
Spatial distribution of public services and residents’ health (China, 2007–2019): **(A)** spatial distribution of public services in China, 2007; **(B)** spatial distribution of public services in China, 2019; **(C)** spatial distribution of residents’ health in China, 2007; **(D)** spatial distribution of residents’ health in China, 2019.

This study mapped the spatial distribution of public services and residents’ health based on data from 2007 to 2019 and the division of China’s administrative regions (Figure 2). The spatial distribution map facilitates a comprehensive understanding of the development level of public services and the health status of residents in China, providing a factual basis for the subsequent research. The differentiated pattern of public services in China does not change significantly over time, and generally presents a pattern of “high in the east and west and low in the central region.” The level of public services in each province does not change much; only a few provinces show an increased or decreased state during the study period.

The spatiotemporal evolution of residents’ health is more evident, with a “high in the east and low in the west” pattern basically in 2007. Over time, the pattern of residents’ health changes significantly in 2019, transitioning from the previous “east–west” to “north–south” pattern, overall presenting a high characteristic in the north and south, and low in the central region. Specifically, residents’ health levels in northern provinces such as Xinjiang, Qinghai, and Gansu improve significantly, but in stronger provinces like Shandong, Liaoning, and Jilin, they weaken; residents’ health levels in the provinces in the central hinterland (Henan, Hubei, Sichuan, Chongqing, etc.) drop significantly; the health levels of residents in many provinces along the southeast coast do not change much.

### Model Specification and Selection

#### Spatial Econometric Model Specification

Grossman [27] first established the health production function to explore the factors influencing residents’ health. As the research deepened, spatial factors became an indispensable consideration in this type of study. Scholars gradually incorporated spatial elements into the model, culminating in the development of various types of spatial econometric models [28].

The selection of an appropriate model among a multitude of spatial econometric models is another significant issue for this study. Public services can influence economic development by promoting the flow of production elements, and the state of economic development can, in turn, impact the progression of public services. Therefore, there is a clear coupling relationship between public services and regional economics. Although residents’ health levels in a province are not directly associated with economic status, an intermediary correlation mechanism might exist. After thorough consideration of the economic attributes of public services and resident health, this study confines the scope of spatial econometric models within the realm of socio-economic studies. According to the various manifestations of spatial effects in models, they can be primarily classified into several types: the spatial autoregressive model (SAR), the spatial error model (SEM) and the spatial Durbin model (SDM). There are other spatial econometric models (such as, SLD, SAC, etc.), but these generally serve as variations of the above models. The specific formulas are as follows:

$${RH}_{it}=\rho \sum_{j=1}^{n}W_{ij}{RH}_{jt}+\beta {PS}_{it}+\alpha_{i}+\gamma_{t}+\mu_{it}$$ (3)

$${RH}_{it}=\beta {PS}_{it}+\alpha_{i}+\gamma_{t}+\nu_{it}\;\nu_{it}=\lambda \sum_{j=1}^{n}W_{ij}\nu_{jt}+\mu_{it}$$ (4)

$${RH}_{it}=\rho \sum_{j=1}^{n}W_{ij}{RH}_{jt}+\beta {PS}_{it}+\theta \sum_{j=1}^{n}W_{ij}{PS}_{jt}+\varphi \sum_{j=1}^{n}W_{ij}{Control}_{it}+\alpha_{i}+\gamma_{t}+\mu_{it}$$ (5)

where ${RH}_{it}$ and ${PS}_{it}$ are the composite score for residents’ health and public services, respectively. ${Control}_{it}$ is the ensemble of other influence variables of ${RH}_{it}$ . The value of ρ expresses the spatial correlation of residents’ health; θ denotes the amount of spatial lag of public services to reveal the mechanism of the effect of public services on other regions; φ reflects the degree of spatial spillover benefits from other explanatory variables; $\alpha_{i}$ and $\gamma_{t}$ are introduced into the model to represent the spatial individual effects and spatial time effects, respectively; and $W_{ij}$ is the ith column of the standardized n × n-dimensional 0–1 spatial weight matrix. $\mu_{it}$ is the random perturbation term, which satisfies the assumptions of zero mean, homoscedasticity and zero covariance.

The economic attributes of public services suggest that biases may arise if studied solely from a geographical perspective. Indeed, spatial and economic connections are maintained between different provinces. Therefore, this study constructed a spatial matrix interwoven with geography and economy (geographical–economic weight matrix) [29]. As the relationship between residents’ health and economic level is not direct, the endogenous association between the two should be eliminated, so this study applied the geographical–economic weight matrix to the spatial lag term of public services and control variables. The formula is as follows:

$$W_{ij}^{d}=\left\{\begin{array}{c} \frac{1}{d_{ij}},i\neq j\\ 0,i=j \end{array}\right.$$ (6)

$$W_{ij}^{\prime }=\left\{\begin{array}{c} \frac{1}{d_{ij}}\frac{per{GDP}_{j}}{per{GDP}_{i}},i\neq j\\ 0,i=j \end{array}\right.$$ (7)

$${RH}_{it}=\rho \sum_{j=1}^{n}W_{ij}{RH}_{jt}+\beta {PS}_{it}+\theta \sum_{j=1}^{n}W_{ij}^{\prime }{PS}_{jt}+\varphi \sum_{j=1}^{n}W_{ij}^{\prime }{Control}_{it}+\alpha_{i}+\gamma_{t}+\mu_{it}$$ (8)

where d represents the geographical distance in terms of latitude and longitude of the provincial center points, and perGDP stands for the *per capita* GDP of each province. To avoid the impact of inflation, *per capita* GDP was deflated using the year 2000 as the base period.

#### Model Evaluation and Selection

The decision to select a specific model was determined based on the following test results.

Lagrange Multiplier (LM) Test: The spatial correlation between residents’ health and public services sets the foundation for spatial econometric research. However, the choice between spatial regression analysis and ordinary least squares (OLS) requires an analysis of spatial dependence in model residuals through the LM test to ascertain if the study must employ spatial regression analysis [30]. Specifically, the LM test is divided into two forms: the LM lag and LM error tests. According to the results (LM–SAR: 333.070***; LM–SEM: 239.454***), both LM tests are significant, implying the need for spatial regression analysis.

Hausman Test: Researchers usually select fixed effects (FE) for regression analysis when dealing with panel data. However, Hausman test results form a rigorous scientific basis for the selected effect. The test result (22.02***) rejects Hausman’s original hypothesis, indicating that for this study’s panel data, FE would provide more rigorous results.

Likelihood Ratio (LR) Test: Unlike the LM test, the main principle of the LR test is to compare the likelihood of alternative models so that the model with the most robust data fit can be selected. Both the LR–SEM (11.35**) and LR–SAR (12.15**) pass the statistical significance test and reject the original hypothesis of model simplification. This result indicates that the SDM is the most suitable spatial model for this study’s data. Moreover, this study reused the LR test to analyze which FE should be selected for the experimental model. The LR test results (FE–spatial: 50.40***; FE–time: 528.77***) reject the hypothesis that single FE are superior to double FE, suggesting that a spatial–temporal double FE should be applied to the spatial model.

In sum, after a series of tests, this study ultimately constructed a spatial Durbin model with double FE on space and time (SDM-stFE) to carry out in-depth research.

### Spatial Model Estimation Results and Analysis

#### Basic Regression Results

This study used the corrected maximum likelihood estimation method for the full sample. In the absence of spatial factors, the coefficient of public services appears negative. This result indicates that the level of public services in a province positively correlates with that of local residents’ health.

The θ (−1.66***) in the SDM-stFE suggests that the benefits of public services manifest more robustly in the spatial dimension. The positive development of public services improves residents’ health levels in a province and boosts residents’ health in neighboring provinces. Regarding the control variables, the spatial lag term results for *per capita* GDP are consistent with W*PSit. However, the urbanization demonstrates negative spatial spillover benefits.

The spatial lag term for residents’ health (W*RHit) demonstrates statistical significance, indicating that residents’ health has spatial autocorrelation across provinces.

#### Spatial Effect Decomposition

When probing the spatial correlation mechanism between public services and residents’ health, the spill-over effect of local public services has a ricochet impact: it affects neighboring provinces, which, in turn, influences the originating province via various channels. This phenomenon, known as the “feedback effect,” introduces bias into the estimation of the spatial benefits of public services in SDM. Regrettably, the estimated coefficients of the model fail to identify the “feedback effect” of the variables. However, the SDM has a unique advantage in that it can decompose the total effect into direct and indirect effects, offering more precise and comprehensible research results.

To provide a more detailed delineation, this study employed a partial differential method to partition the effect of public services on residents’ health into direct, indirect, and total effects [31–33]. The general form is:

$$Y_{t}={\left(I-\delta W\right)}^{-1}\alpha_{t}\tau_{N}+{\left(I-\rho W\right)}^{-1}\left(X_{t}\beta +WX\theta \right)+{\left(I-\rho W\right)}^{-1}\varepsilon$$ (9)

The matrix of the partial derivative differential equation is:

$$\left[\frac{\partial y}{\partial X_{ik}}\cdots \frac{\partial y}{\partial X_{NK}}\right]=\left[\begin{array}{ccc} \frac{\partial y_{1}}{\partial X_{ik}} & \cdots & \frac{\partial y_{1}}{\partial X_{NK}}\\ \vdots & \vdots & \vdots \\ \frac{\partial y_{N}}{\partial X_{ik}} & \cdots & \frac{\partial y_{N}}{\partial X_{NK}} \end{array}\right]={\left(I-\rho W\right)}^{-1}\left[\begin{array}{ccc} \begin{array}{cc} \beta_{k} & w_{12}\theta_{k} \end{array} & \cdots & w_{1N}\theta_{k}\\ \begin{array}{cc} w_{21}\theta_{k} & \beta_{k} \end{array} & \ldots & w_{2N}\theta_{k}\\ \begin{array}{cc} \begin{array}{c} \vdots \\ w_{N1}\theta_{k} \end{array} & \begin{array}{c} \vdots \\ w_{N2}\theta_{k} \end{array} \end{array} & \begin{array}{c} \vdots \\ \ldots \end{array} & \begin{array}{c} \vdots \\ \beta_{k} \end{array} \end{array}\right]$$ (10)

According to the indirect effects results, public services indeed exhibit a fairly significant positive spatial spillover effect. Liang, in exploring the relationship between public health services and residents’ health in China, found that public health services had a noticeable spatial spillover effect [34]. By extending the object of this study to overall public services, consistent findings were obtained.

After reviewing the literature, an initial analysis suggested that public services exerted their spatial spillover effect through two avenues. First, there are close ties between different provinces in China. Under the guidance of the central government, a regional cooperation pattern has formed based on resource sharing. As such, provinces with higher levels of public services, such as in education and healthcare, will have a “radiating” effect, promoting public service development and the improvement of residents’ health in neighboring provinces [35]. In this context, the population, as a unique resource, moves between regions and aids the “spread” of superior public services to other areas. Second, due to the strong coupling relationship between public services and economic development, the spillover effect of the economy may stimulate the benefits of public services to neighboring regions [36].

### Robustness Test

To ensure that this study’s results would be universal and reliable rather than exceptional, this study used the adjusted Mazziotta-Pareto index (AMPI) to rebuild the composite indicators and observe the changes that could occur in the results [37]. During the processing of AMPI, penalties are given to imbalanced indicator values to encourage the selection of more balanced units when the indicator values are equal. Therefore, for residents’ health, the increase in the value of the final composite index represents an actual improvement in residents’ health levels [38].

The comprehensive indicator results obtained from the AMPI are used to reconduct the spatial regression analysis. The results show that the nature and degree of the spatial impact of public services on neighboring provinces remain consistent with the previous results, with considerable spatial spillover benefits of public services. After replacing the method of constructing the comprehensive index and reconducting the analysis, the obtained results are consistent with the previous results, demonstrating the reliability of this study’s empirical findings. Table 3 presents all the results of the basic regression, spatial effect decomposition, and robustness checks.

**TABLE 3:** Full sample spatial regression results and robustness test findings (China, 2007–2019).

Variable	SDM (entropy method)	SDM (AMPI)	Direct effect	Indirect effect	Total effect
Public Services	−0.766*** (0.1231)	−0.0450 (0.1284)	−0.835*** (0.1350)		−3.525*** (0.9147)
Logarithm of GDP per Capita	−0.00516 (0.0248)	−1.1496 (1.3558)	−0.0116 (0.0240)		−0.251*** (0.0529)
Urbanization	−0.241 (0.1355)	−20.390*** (7.2205)	−0.163 (0.1279)		2.958** (0.9442)
Human Capital	−0.0123 (0.1230)	11.509* (6.4392)	0.00845 (0.1250)		0.906 (0.5558)
W*PSit	−1.661** (0.5965)	0.9675** (0.4930)		−2.691** (0.8649)	
W*Logarithm of GDP per Capita	−0.168*** (0.0456)	−3.0931 (2.9084)		−0.240*** (0.0586)	
W*Urbanization	2.281*** (0.6630)	2.7048 (25.255)		3.121** (0.9622)	
W*Human Capital	0.648 (0.4069)	−8.9843 (21.117)		0.898 (0.5537)	
W*RHit	0.313*** (0.0638)	0.4663*** (0.0519)			
Sigma2	0.00119*** (0.0001)	3.5550*** (0.0519)			
*R* ^2^	0.1760	0.3515			
N	390	390			

Note: Standard errors in parentheses; ***, **, and * indicate 1%, 5%, and 10% significant levels, respectively.

### Endogeneity Test

During the research process, unobservable factors may have introduced bias into the results, leading to inevitable endogeneity problems. To overcome the endogeneity problem, this study conducted a one-period time lag treatment for residents’ health, incorporating the time-lagged term of residents’ health as an instrumental variable into the model:

$${RH}_{it}=\rho \sum_{j=1}^{n}W_{ij}{RH}_{jt}+{\sigma RH}_{jt-1}+\beta {PS}_{it}+\theta \sum_{j=1}^{n}W_{ij}^{\prime }{PS}_{jt}+\varphi \sum_{j=1}^{n}W_{ij}^{\prime }{Control}_{it}+\partial$$ (11)

$$\partial =\alpha_{i}+\gamma_{t}+\mu_{it}$$ (12)

When decomposing spatial benefits, the Mundlak regression was used to separate them into short-term and long-term effects, further ensuring the accuracy of the results [39]. After incorporating the instrumental variables into the model, the benefits of each variable do not undergo significant changes. However, the endogeneity test results reveal an important issue: public services only exhibit a spillover effect in the short term. Specifically, the benefits of public services in promoting residents’ health in neighboring provinces are effective only in the short term, with no significant long-term benefits. Table 4 specifically presents the results of the endogeneity test.

**TABLE 4:** Endogenous estimation results (China, 2007–2019).

Variable	Basic benefit	Benefit decomposition	
		Short term	Long term
Residents’ Health (L1)	0.715*** (0.0383)		
Public Services	−0.449*** (0.1013)		
Logarithm of GDP per Capita	0.0226 (0.0202)		
Urbanization	−0.0461 (0.1101)		
Human Capital	−0.0955 (0.0942)		
W*RHit	0.350*** (0.0580)		
Direct- PS		−0.514*** (0.1048)	−0.7586 (12.778)
Direct- GDP per Capita		0.0226 (0.0194)	0.1594 (0.6238)
Direct- Urbanization		−0.0028 (0.1008)	−1.2251 (9.9663)
Direct- Human Capital		−0.0393 (0.0915)	−1.4011 (11.004)
Indirect- PS		−2.125*** (0.7154)	34.439 (355.28)
Indirect- GDP per Capita		−0.123** (0.0569)	1.4670 (17.774)
Indirect- Urbanization		2.099*** (0.7789)	−27.768 (285.56)
Indirect- Human Capital		2.467*** (0.4869)	−26.628 (315.95)
Total- PS		−2.640*** (0.7470)	33.680 (367.36)
Total- GDP per Capita		−0.1005* (0.0538)	1.6263 (18.383)
Total- Urbanization		2.096*** (0.7651)	−28.994 (295.37)
Total- Human Capital		0.428*** (0.5028)	−28.029 (326.78)

Note: Standard errors in parentheses; ***, **, and * indicate 1%, 5%, and 10% significant levels, respectively.

## Discussion

### Conclusion

This study established a SDM through spatial autocorrelation, Hausman, LM and LR tests, and based on panel data from 30 provinces in China from 2007 to 2019, the link between residents’ health and public services was explored. The findings are as follows. 1) Both residents’ health and public services exhibit a high degree of spatial autocorrelation but show a downward trend over time. Specifically, the eastern region consistently maintains a very high level of residents’ health and a high degree of provincial clustering. Residents’ health levels in most northern provinces are improving annually, while most central provinces show the opposite state. The clustering degree of public services in each province does not change much, and there is a clear divide between the different cluster types [40]. 2) The positive spatial spillover effect of public services is a core finding of this study. The enhancement of public service capability positively affects local residents’ health and boosts residents’ health in neighboring provinces. The regional cooperation mechanism for resource sharing has been constructed among various Chinese provinces, and population mobility provides support for the dissemination of superior public services, underpinning the spillover influence of public services in the health domain [41]. 3) Per capita GDP also has a spatial spillover effect that elevates residents’ health, which, to a certain extent, verifies the spillover property of the economy. Combining the coupling relationship between public services and the economy, the further analysis shows that the spillover effect of public services is also supported by economic spillover. 4) Upon integrating the time-lagged items as instrumental variables into the model for endogeneity testing, the spatial spillover effect of public services is only seen in the short term. In a given province, residents’ health levels will be influenced by local and external public services in the short term; in the long term, the impact of local public services is more pronounced.

### Limitations and Improvements

Compared to the extant research’s focus on specific public service types, this study used spatial econometric analysis to explore public services as a whole from a more comprehensive perspective. The core result (the positive spatial spillover effect of public services) is an innovative finding that enriches the current network system of results for public services. However, this study encountered objective limitations when collecting panel data. Under the influence of factors such as the time span and policy reforms, some data were unable to meet the needs of this study, compelling us to abandon certain evaluation indicators.

Meanwhile, few studies have incorporated geospatial factors into public service and health research. However, for countries with large land areas, the influence of geospatial factors on such research cannot be ignored, as the spatial benefits of public services go far beyond even those that have traditional statistical significance [42]. With the deepening of the research and the development of spatial statistical technology, the future studies on public services and residents’ health will become more standardized, as follows. 1) The index system of public services and population health will be optimized, and the comprehensive indicator framework will better reflect the actual levels of public services and residents’ health. 2) Spatial geographical factors will become a necessary variable in the future research. 3) The serious income inequality problem worldwide and its integrated factors, when combined with spatial geography, will become an important perspective for the subsequent population health studies [43]. 4) Considering the pronounced lagged nature of public services and associated policies, it could be beneficial for the future research to leverage the difference-in-differences approach to evaluate the impacts of various policies implemented by China’s central government.
